# Comparison of *Helicobacter pylori* eradication regimens in patients with end stage renal disease 

**Published:** 2018

**Authors:** Mohammadreza Seyyed Majidi, Peyman Sanjari Pirayvatlou, Majid Rajabikashani, Mona Firoozabadi, Seyed ali Seyed Majidi, Jamshid Vafaeimanesh

**Affiliations:** 1 *Golestan Research Center of Gastroenterology and Hepatology (GRCGH), Golestan University of Medical Sciences (GOUMS), Gorgan, Golestan Province, Iran*; 2 *Gastroenterology &Hepatology Research Center. Qom Univercity of Medical Séances, Qom, Iran*; 3 *Gastroentrology and Liver Disease Research Center, Iran University of Medical Sciences, Tehran, Iran *

**Keywords:** End Stage Renal Disease, Eradication therapy, *Helicobacter pylori*

## Abstract

**Aim:**

The aim of this study was to compare the *Helicobacter pylori* (HP) eradication regimens in patients with end stage renal disease.

**Background:**

In patients undergoing hemodialysis, the pathologic changes seen in the stomach may be the result of high serum levels of gastrin, delayed gastric emptying or HP infection.

**Methods:**

Our study was a randomized clinical trial in which 120 patients with ESRD (Patients who undergo hemodialysis) confirmed HP infection, were divided to four groups having 2-week eradication regimens; Group I: LCA (lansoprazole 30 mg-BD,clarithromycin 250 mg-BD, amoxicillin 500 mg-BD), Group II: LCM (lansoprazole 30 mg-BD,clarithromycin 250 mg-BD, metronidazole 500 mg-BD), Group III: LCAM (lansoprazole 30 mg-BD,clarithromycin 250 mg-BD,amoxicillin 500 mg-BD, metronidazole 500 mg-BD) and Group IV: Sequential (lansoprazole 30 mg-BDfor two weeks; first week: amoxicillin 500 mg-BD and second week: clarithromycin 250 mg-BD, metronidazole 500 mg-BD).6 weeks after treatment, Urea Breath Test (UBT) was performed for all patients.

**Results:**

The mean age of patients was 43.1±11.2 years. 55.8% of patients were male. The success rates of HP eradication in 4 groups were76.7%, 70%, 90% and 90%, respectively. HP eradication rates were not statistically different among the regimens (p=0.11). There were not significant differences among the groups regarding demographic and anthropometric variables.

**Conclusion:**

The results showed there was no significant difference between the success rates of HP eradication regimens for ESRD patients. According to approved regimen for 90% eradication rate, with a lower number of medications and given the less risk of side effects and drug interactions, the sequential regimen is the best.

## Introduction

*Helicobacter pylori *(HP) is a Gram-negative bacterium and its special microbiological feature contains urease enzyme that allows bacterial colonization in the gastric antrum. Several studies have shown that HP infection causes complications such as chronic gastritis, peptic ulcer disease; mucosa associated lymphoid tissue (MALT) lymphoma and gastric cancer. HP has about 50% of the adult population involved, despite the fact that only 20%of these patients are symptomatic. Several factors, including geography, culture, age and socio-economic factors involved in the development of HP infection. The prevalence of HP in the United States and other developed countries is about 30% and in developing countries is more than 80%. Except for age, other major risk factor is low socio-economic level of society (1-4).

The success of eradication treatment of HP decreased from 90% in 1990 to fewer than 60 percent. The main cause of treatment failure described as resistance to antimicrobial drugs, especially to clarithromycin. Now it has been found that bacteria can make output channels in the cell wall to exorcise clarithromycin and prevents it from binding to ribosomes. Cell wall destruction by amoxicillin prevents creating these channels and improves clarithromycin performance (5-11). Almost 60% drop in eradication with standard triple therapy will occur when there is a resistance to clarithromycin, so that the use of standard triple therapy is limited to regions where there is resistance to clarithromycin about 15 to 20%. The physician should prescribe a treatment regimen with power more than 85-90%. Some researchers have suggested that increasing the duration of treatment will result in a higher rate of eradication (12). Updated treatment strategies in HP infection presented in the guidelines of the Toronto Consensus Group (2016). These suggest to prolong eradication therapy up to 14 days, replacing the old triple therapy with a quadruple therapy based on proton pump inhibitor (PPI), bismuth, metronidazole, and tetracycline for most of the patients, or as an alternative quadruple therapy without bismuth, based on the use of PPI, amoxicillin, metronidazole, and clarithromycin (13). The prevalence of resistance to antibiotics in several countries is different. Due to the geographical diversity, determining the best HP treatment should be based on the geographic distribution of antibiotic resistance. Another important factor is CYP2C19 polymorphism responsible for biotransformation of many drugs called proton-pump inhibitors (PPI). The effect of age and gender on the success of treatment is uncertain (14,15). 

The gastrointestinal symptoms, common in patients with renal function impairment, constitute an important component of the uremic syndrome. In patients undergoing hemodialysis, the pathologic changes seen in the stomach may be the result of high serum levels of gastrin, delayed gastric emptying or HP infection (16,17). It seems that HP infection of the gastric mucosa plays a central role in causing several gastroduodenal lesions. The hypothesis that a high level of urea in the gastric mucosa in patients with advanced renal failure might predispose them to HP infection is derived from the notion that HP urease converts urea to ammonia and raises the local gastric pH and enhances the survival of the organism (18). It seems that in patients with advanced kidney failure, high serum levels of urea, is the main factor to HP colonization of the stomach and upper gastrointestinal mucosal inflammation. A higher rate of duodenal and gastric mucosal lesions in uremic patients with HP infection compared to those with normal renal function may be developed due to high levels of serum urea, anemia and blood flow levels stomach. ESRD patients waiting for kidney transplants need to get effective treatment for eradication of HP, because they weaken the immune system after transplantation will aggravate the side effects of HP infection (19-22).

We used different treatment regimens in this study such as conventional triple and quadruple therapy treatments without bismuth (due to its toxic effects in renal failure) to identify the effectiveness of each of these regulations and determine the best treatment regimen. 

## Methods

A prospective randomized clinical trial study was conducted on 120 consecutive HP infected patients with ESRD between June 2014 and September 2015. All of them were referred to our academic hospital in Gorgan (north of Iran). Exclusion criteria were previous HP eradication, consumption of aspirin, non-steroid anti-inflammatory drugs (NSAIDs), proton pump inhibitors (PPI), warfarin, bismuth preparations or antibiotics during the last 8 weeks. Gastroscopy was done using a video scope (Olympus GIF-XQ260, Japan) and two specimens were obtained from the antrum HP infection was diagnosed by histopathological examination. This research was approved by the Ethical Committee in Golestan University of Medical Sciences. Informed consent was obtained from all patients. 

The Patients' demographic characteristics such as age and gender were recorded. Height and weight were measured. Based on measured height and weight data of patients, BMI (Body Mass Index = BMI) was calculated for each of them.

Patients were divided into four groups of 30 members:

1- Group I: Triple Therapy (LCA) (lansoprazole 30 mg-BD, clarithromycin 250 mg-BD, amoxicillin 500 mg-BD for 2 weeks)

2- Group II: triple therapy (LCM) (lansoprazole 30 mg-BD, clarithromycin 250 mg-BD, metronidazole 500 mg-BD for 2 weeks)

3- Group III: quadruple therapy (LCAM) (lansoprazole 30 mg-BD, clarithromycin 250 mg-BD, amoxicillin 500 mg-BD, metronidazole 500 mg-BD for 2 weeks)

4- Group IV: sequential treatment (Sequential) (lansoprazole 30 mg-BD for two weeks; first week: amoxicillin 500 mg-BD and second week: clarithromycin 250 mg-BD, metronidazole 500 mg-BD).

A 13C-urea breath test was performed for eradication assessment 6 weeks after completion of the treatment. The collected data inserted and encoded with statistical software SPSS-18 and were analyzed. To describe the data, frequency, percentage, mean and standard deviation were used. To compare the age, sex, height, weight and BMI between the groups studied Chi-square (2χ) and analysis of variance (ANOVA) and to compare the eradication of HP and UBT test results between the groups studied, chi-square (χ2) was used. 

## Results

120 ESRD patients with proven HP infection in 4 groups of 30 patients with different treatment regimens were examined. The overall average age of the patients was 43.1 ± 11.2 years (range 22-79 years). 67 (55.8%) of them were male. In terms of BMI, 12(10%) were lower than normal (Underweight), 55 (45.8%) normal, 34 (28.3%) overweight, 18 (15 %) obese and 1 patient (0.8%) had morbid obesity. Analytical review of demographic and anthropometric variables showed that the groups studied in terms of age, sex and BMI, had no statistically significant difference (Table 1).

The results of UBT after treatment were negative in 98 patients (81.7%).The success rates of HP eradication in 4 groups were 76.7%, 70%, 90% and 90% respectively. HP eradication rates were not statistically different among the regimens (p=0.11) but 90% eradication rate was achieved in sequential and LCAM groups. There were not significant differences among the groups regarding demographic and anthropometric variables. No significant side effects following the treatment regimens used in ESRD patients has been reported.

## Discussion

Gastrointestinal symptoms in patients with chronic renal failure are abundant. Erosive gastritis and peptic ulcer are the most common upper gastrointestinal disorders and maybe the factors such as an increase in gastrin and stomach acid, increased parathyroid hormone, decreased mucosal resistance and delayed gastric emptying are the main causes (22). Maintaining PPI plasma concentrations in high levels and antibiotics even on low-dose is a notable point that should be considered in HP eradication of hemodialysis patients.

As Ehsani-Ardakani and colleagues have stated: Half-dose triple-therapy with clarithromycin, amoxicillin and omeprazole is as effective as full-dose triple-therapy to eradicate the Hp in patients with ESRD (23). Because of the immune deficiency in hemodialysis patients, the possibility of infections caused by antibiotic-resistant strains at the time of taking drugs exists. 36.4% of ESRD patients are infected by clarithromycin resistant strain that is significantly more than the control group (24). Patients with hemodialysis that have immunodeficiency may suffer hemorrhagic lesions caused by HP infection and usually symptomatic therapy is not effective in these patients. Etiologic approach to infection and its treatment prevents upper gastrointestinal bleeding and iron deficiency anemia. The treatment can also prevent intermittent bleeding from erosive gastritis, erosive duodenitis and peptic ulcer caused by persistent infection (25). Some studies reported the prevalence of HP infection in patients with renal failure similar to normal population (26-28). In other studies, the prevalence of HP infection in patients with impaired kidney function is less than the normal population that the cause has been attributed to a protective role of high urea concentrations or acid-reducing drugs and antibiotics (29,30).

**Table 1:** Baseline characteristics of study subjects in four groups

Character	Groups				Total (n=120)	*P* *value*
	LCA (n=30)	LCM (n=30)	LCAM (n=30)	Sequential (n=30)		
Age (years)	43.9±10.9	44.2±13.4	42.5±10.4	41.7±10.0	43.1± 11.2	0.67
Gender (F/M)	13/17	13/17	13/17	14/16	53/67	0.98
BMI (Kg/m^2^)	24.2±4.4	25.4±5.6	25.0±5.1	24.5±5.6	24.6±5.2	0.73

LCA = Lansoprazole, Amoxicillin and Clarithromycin; LCM = lansoprazole, clarithromycin and metronidazole; LCAM = lansoprazole, clarithromycin, amoxicillin and metronidazole

**Table 2:** *Helicobacter pylori* eradication rates of study subjects in four groups

Character	Groups				*P* *value*
	LCA (n=30)	LCM (n=30)	LCAM (n=30)	Sequential (n=30)	
Eradication rate (%)	23 (76.7%)	21 (70%)	27 (90%)	27 (90%)	0.11

LCA = Lansoprazole, Amoxicillin and Clarithromycin; LCM = lansoprazole, clarithromycin and metronidazole; LCAM = lansoprazole, clarithromycin, amoxicillin and metronidazole

In our study, no significant differences among four HP eradication regimens in ESRD patients has been detected. Considering the high potency and effectiveness of sequential regimen with 90% HP eradication success and lesser drug prescription, as respects drug side effects on ESRD patients and drug interactions, we offer this regimen to prescribe for ESRD patients

## Conflict of interests

The authors declare that they have no conflict of interest.
